# Impulse Control Disorder Behaviors in Dystonia

**DOI:** 10.1002/mds.29230

**Published:** 2022-09-28

**Authors:** Annu Huovinen, Elina Jaakkola, Juho Joutsa

**Affiliations:** ^1^ Clinical Neurosciences University of Turku Turku Finland; ^2^ Turku Brain and Mind Center University of Turku Turku Finland; ^3^ Neurocenter Turku University Hospital Turku Finland

Impulse control disorders (ICDs) are common in patients with Parkinson's disease (PD) and strongly linked with dopaminergic medication.^1^ Given that dopamine agonists (DAs) are the main risk factor, ICDs have only been studied in disorders that are treated with these medications. Restless legs syndrome, prolactinoma, and fibromyalgia have all been shown to have an increased risk for ICDs compared with healthy controls (HC).^2^ However, patients without DAs can also develop ICDs, and there are several case reports of ICDs in other chronic neurological conditions without dopaminergic medications,^1^, ^2^ suggesting that ICDs could be a relevant neuropsychiatric symptom also in other chronic neurological disorders.

Dystonia is a chronic movement disorder that is not considered a dopamine‐dependent nor is treated with dopaminergic medications (with rare exceptions). Similar to PD, though, dystonia is associated with a high burden of neuropsychiatric comorbidity, including for example, depression, anxiety, and sleep disorders, which have been shown to increase the risk of substance use disorders in patients with dystonia.^3^, ^4^ These neuropsychiatric symptoms are also common comorbidities of ICDs.^1^ However, ICDs in dystonia have not been studied. The aim of this study was to investigate if dystonia is associated with ICDs by directly comparing ICD prevalence in dystonia with that of HC and patients with PD with DAs (PD + DA) and without DAs (PD − DA).

ICDs were evaluated using the Questionnaire for Impulsive‐Compulsive Disorders (QUIP) using a survey e‐mailed personally to the members of the Finnish Movement Disorders Association, as described previously.^5^ Altogether, 98 patients with dystonia, 498 patients with PD (317 PD + DA, 181 PD − DA) and 119 HC provided sufficient information to be included in the study (Table S1). Full methodological details can be found in the Supplementary Material.

Prevalence of positive screens for ICDs in dystonia (20.4%) did not differ from HC (21.8%, *P* = 0.87) or PD − DA (26.0%, *P* = 0.38), but was significantly lower than in PD + DA (33.8%, *P* = 0.02) Fig. 1, Table S1). In the multiple regression analysis, ICDs were independently associated with PD + DA compared with HC (odds ratio [OR], 1.70 [95% confidence interval, CI, 1.02–2.84]; *P* = 0.04), younger age (OR, 1.51 [95% CI, 1.26–1.80] for 10‐year decrease; *P* < 0.001), and male sex (OR, 2.23 [95% CI, 1.56–3.20]; *P* < 0.001), but not with dystonia (OR, 0.92 [95% CI, 0.48–1.78]; *P* = 0.80) (Table S2–S4).

**FIG 1:**
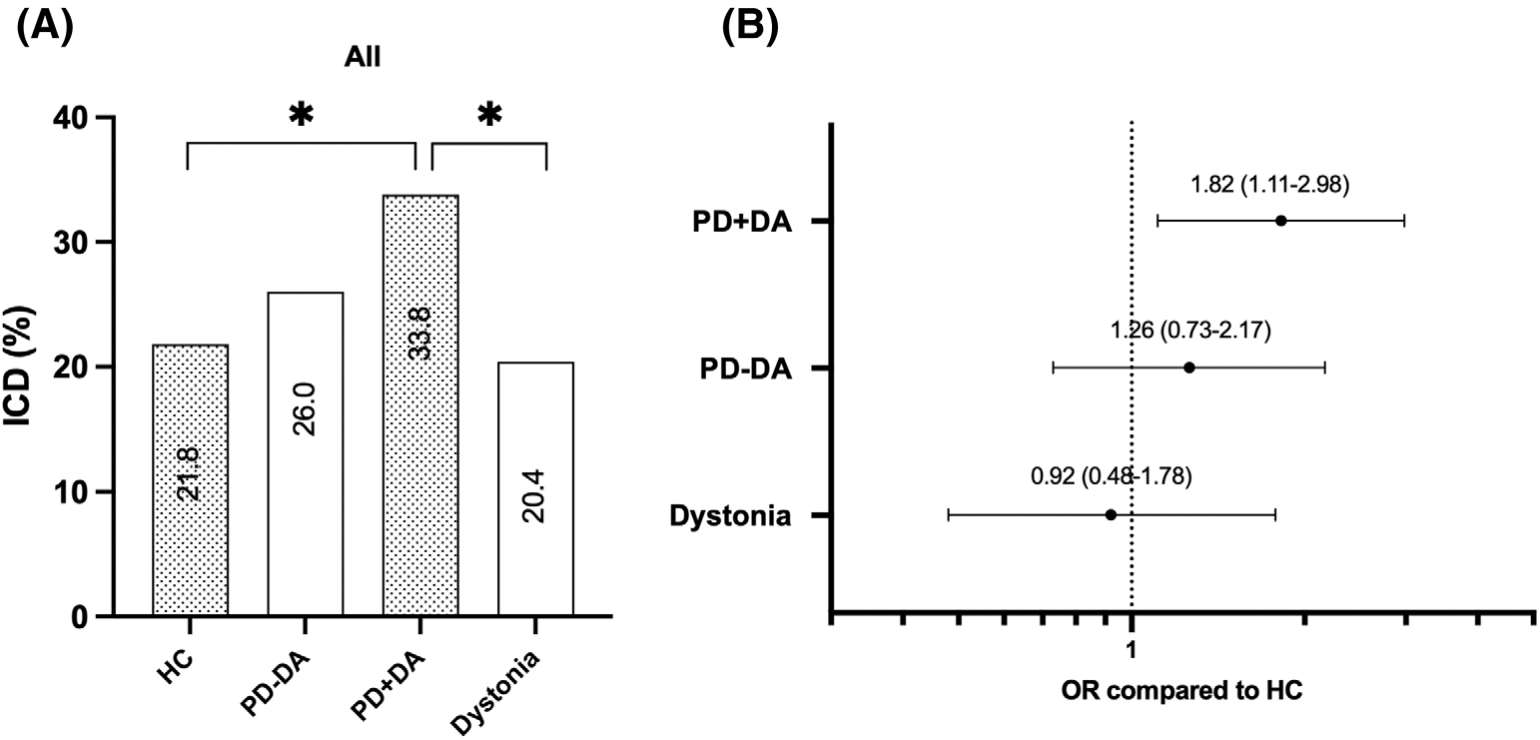
Impulse control disorders (ICDs) in patients with dystonia and Parkinson's disease (PD). (**A**) Prevalence of ICDs estimated using the Questionnaire for Impulsive‐Compulsive Disorders (QUIP) in healthy controls (HC), PD without dopamine agonists (PD − DA), PD with dopamine agonists (PD + DA), and dystonia. *P<0.05 (**B**). Odds ratio (OR) (95% confidence interval) of ICDs in PD + DA, PD − DA, and dystonia compared with HC (univariate regression analysis).

Our results shown that the prevalence of ICDs in dystonia is comparable with HC and PD − DA, but less common than in patients with PD + DA. Our findings support the view that ICDs are linked with dopaminergic treatment, and not with chronic neurological conditions in general.

The limitations of this study include the use of QUIP, which as a screening instrument is associated with false positives. In addition, the sample size could be considered low for estimating the prevalence of ICDs in dystonia. However, our aim was not to estimate the actual prevalence of ICDs but to investigate whether dystonia is associated with increased ICD risk. As the proportion of patients with ICDs in dystonia was slightly lower than in PD − DA or controls, it is highly unlikely that the lack of detecting an increased ICD risk in dystonia would be a sample size issue.

## Author Roles

(1) Research Project: A. Conception, B. Organization, C. Execution; (2) Statistical Analysis: A. Design, B. Execution, C. Review and Critique; (3) Manuscript Preparation: A. Writing of the First Draft, B. Review and Critique.

A.H.: 1A, 1C, 2A, 2B, 3A

E.J.: 1A, 1C, 2A, 2B, 2C, 3B

J.J.: 1A, 1B, 1C, 2A, 2C, 3A

## Financial Disclosures

Annu Huovinen has received a grant from the Finnish Parkinson Foundation. Elina Jaakkola has received a grant from the Finnish Parkinson Foundation. Juho Joutsa has received lecturer honoraria from Lundbeck; research grants from the Finnish Medical Foundation, Sigrid Juselius Foundation, Instrumentarium Research Foundation, Finnish Foundation for Alcohol Studies, University of Turku, private donation, and Turku University Hospital (Finnish governmental research funding); and conference travel support from Abbvie.

## Supporting information


**Appendix S1** Supporting Information

## Data Availability

The data can be shared upon reasonable request, subject to institutional and national regulations
