# Supplementary material for: Impulse Control Disorder Behaviors in Dystonia
Source: Mov Disord. 2022 Sep 28;37(12):2460–1. doi: 10.1002/mds.29230 (PMC10087985; doi:10.1002/mds.29230)
Supplement: Supplementary file 1 — Appendix S1 Supporting Information [file MDS-37-2460-s001.docx]

**Methods**

***Participants***

The questionnaire was sent to the members of the Finnish Movement Disorders Association via email, which is currently the primary communication method of the association^1^. The answers were collected using REDCap electronic data capture tool hosted at University of Turku ^2, 3^. All data was collected between November 2019 and December 2020.

Altogether 892 subjects took part in the study. Subjects with coexisting PD and dystonia (n=7), with insufficient medication information (n=10) and incomplete questionnaire data (n=160) were excluded, resulting in 715 subjects that were included in the analyses. In the included sample, there were 98 patients with dystonia, 498 patients with PD (317 with dopamine agonist medication (PD+DA) and 181 without dopamine agonists (PD-DA)) and 119 healthy controls.

The study protocol was approved by Turku University Hospital Clinical Research Services Board. The need for separate ethics board review was waived. Written informed consent was obtained from all study participants and the study was conducted according to the principles of the declaration of Helsinki.

***Questionnaires***

ICDs were evaluated using the Questionnaire for Impulsive-Compulsive Disorders in Parkinson’s Disease (QUIP) ^4^. In addition, demographic and clinical data including age, gender, education, year of dystonia/PD symptom onset, year of dystonia/PD diagnosis, dystonia type, current medications and history of neurosurgical treatments were collected.

QUIP data was analyzed as described in our previous studies ^1, 5-7^. Cut-off scores for QUIP were at least two positive answers in QUIP sub-sections of gambling disorder and hypersexuality, and at least one positive answer in QUIP sub-sections of compulsive shopping and binge eating, according to the original validation study ^4^.

If more than 20% of the answers were missing in QUIP subsection (more than one out of five) the score of that subsection was considered as missing value in the analyses. If only one of the answers was missing in a QUIP subsection, the missing value was interpreted as no / zero points. If one or more of the subsections was positive, the subject was considered to have positive screen for ICDs. If two or more of the subsections were positive, the subject was considered to have positive screen for multiple ICDs.

***Statistics***

The statistical analyses were performed using SPSS Statistics version 26 (IBM Corp., New York, USA). Fisher’s exact test or one-way ANOVA with Bonferroni correction for *post hoc* tests were used to compare the demographical/clinical factors and ICDs between the groups. Binary logistic regression analyses were used to control for potential confounding variables and investigate if the patient groups are independently associated with ICDs compared to controls. Only variables with significant association in univariate analyses were included in the corresponding multiple regression analysis. HC was used as the control group to calculate the odds ratios (ORs) with their 95% confidence intervals (95% C.I.). P values less than 0.05 were considered significant.

**References**

1. Jaakkola E, Huovinen A, Kaasinen V, Joutsa J. No Change in Prevalence of Impulse Control Disorder Behaviors in Parkinson's Disease During the Last Decade. Mov Disord 2021;36(2):521-523.

2. Harris PA, Taylor R, Thielke R, Payne J, Gonzalez N, Conde JG. Research electronic data capture (REDCap)--a metadata-driven methodology and workflow process for providing translational research informatics support. J Biomed Inform 2009;42(2):377-381.

3. Harris PA, Taylor R, Minor BL, et al. The REDCap consortium: Building an international community of software platform partners. J Biomed Inform 2019;95:103208.

4. Weintraub D, Hoops S, Shea JA, et al. Validation of the questionnaire for impulsive-compulsive disorders in Parkinson's disease. Mov Disord 2009;24(10):1461-1467.

5. Joutsa J, Martikainen K, Vahlberg T, Kaasinen V. Effects of dopamine agonist dose and gender on the prognosis of impulse control disorders in Parkinson's disease. Parkinsonism Relat Disord 2012;18(10):1079-1083.

6. Joutsa J, Martikainen K, Vahlberg T, Voon V, Kaasinen V. Impulse control disorders and depression in Finnish patients with Parkinson's disease. Parkinsonism Relat Disord 2012;18(2):155-160.

7. Jaakkola E, Kaasinen V, Siri C, et al. Impulse control disorders are associated with multiple psychiatric symptoms in Parkinson's disease. J Parkinsons Dis 2014;4(3):507-515.

**Supplementary table 1. Demographical data and prevalence of ICDs. The values are mean (SD) or n (%).**

| **All (n=715)** |
| --- |

|  | HC  (n=119) | PD+DA  (n=317) | PD-DA  (n=181) | Dystonia  (n=98) | F value | P value^1^ | P value^1^  HC vs PD+DA | P value^1^  HC vs  PD-DA | P value^1^  HC vs Dys | P value^1^  PD+DA vs  PD-DA | P value^1^  PD+DA vs Dys | P value^1^  PD-DA vs Dys |
| --- | --- | --- | --- | --- | --- | --- | --- | --- | --- | --- | --- | --- |
| **Age** | 65.22  (12.26) | 65.33  (8.20) | 69.44  (7.70) | 55.76  (12.45) | 43.70^2^ | <**0.001^2^** | 1.00^2^ | **0.001^2^** | **<0.001^2^** | **<0.001^2^** | **<0.001^2^** | **<0.001^2^** |
| **Male gender** | 45 (37.8) | 166 (52.4) | 110 (60.8) | 15 (15.5) |  | **<0.001^1^** | **0.007^1^** | **<0.001^1^** | **<0.001^1^** | 0.08^1^ | **<0.001^1^** | **<0.001^1^** |
| **Any ICD** | 26 (21.8) | 107 (33.8) | 47 (26.0) | 20 (20.4) |  | **0.02** | **0.02** | 0.49 | 0.87 | 0.09 | **0.01** | 0.38 |
| **Multiple ICDs** | 6 (5.0) | 31 (9.8) | 16 (9.0) | 5 (5.1) |  | 0.27 |  |  |  |  |  |  |
| Gambling | 2 (1.7) | 17 (5.4) | 8 (4.5) | 2 (2.1) |  | 0.28 |  |  |  |  |  |  |
| Sex | 9 (7.6) | 63 (19.9) | 32 (17.8) | 5 (5.2) |  | **<0.001** | **0.001** | **0.02** | 0.58 | 0.64 | **<0.001** | **0.003** |
| Shopping | 10 (8.4) | 26 (8.2) | 12 (6.7) | 9 (9.2) |  | 0.88 |  |  |  |  |  |  |
| Eating | 14 (11.8) | 37 (11.7) | 13 (7.3) | 10 (10.2) |  | 0.42 |  |  |  |  |  |  |

**^1^**Fisher’s exact test, **^2^**One-way ANOVA with Bonferroni correction for post-hoc tests.

ICD = Impulse control disorder, HC = Healthy controls, PD+DA = Parkinson’s disease with dopamine agonist medication,

PD-DA = Parkinson’s disease without dopamine agonist medication

Note that the number of subjects included in each analysis varies according to how many subjects provided sufficient data.

**Supplementary table 2.** Univariate and multiple binary logistic regression analyses.

|  | Univariate |  | | | Multiple | |  |  |  |
| --- | --- | --- | --- | --- | --- | --- | --- | --- | --- |
|  | OR (95% CI) | | P value | | OR (95% CI) | | P value | n^1^ |  |
| *Any ICD* |  | |  | |  | |  | 712 |  |
| Age | 1.29 (1.11-1.50) | | **0.001** | | 1.51 (1.26-1.80) | | **<0.001** |  |  |
| Gender (male) | 2.07 (1.48-2.89) | | **<0.001** | | 2.23 (1.56-3.20) | | **<0.001** |  |  |
| Dg (PD+DA)    Dg (dys)    Dg (PD-DA) | 1.82 (1.11-2.98)  0.92 (0.48-1.78)  1.26 (0.73-2.17) | | **0.02**  0.80  0.42 | | 1.70 (1.02-2.84) | | **0.04** |  |  |
| *Any ICD – Only women* |  | |  |  | |  | | 378 | |
| Age | 1.44 (1.17-1.78) | | **<0.001** | | 1.44 (1.17-1.78) | | **<0.001** |  |  |
| Dg (PD+DA)    Dg (dys)  Dg (PD-DA) | 1.69 (0.84-3.40)  1.23 (0.55-2.74)  0.77 (0.31-1.89) | | 0.14  0.62  0.57 | |  | |  |  |  |
| *Any ICD – Only men* |  | |  |  | |  | | 345 | |
| Age | 1.40 (1.09-1.81) | | **0.009** | | 1.40 (1.09-1.81) | | **0.009** |  |  |
| Dg (PD+DA)    Dg (dys)  Dg (PD-DA) | 1.67 (0.82-3.41)  0.62 (0.15-2.55)  1.25 (0.59-2.66) | | 0.16  0.50  0.57 | |  | |  |  |  |

OR=Odds ratio

^1^Number of patients in the multiple analysis

**Supplementary Table 3. Only women (n=378). The values are mean (SD) or n (%).**

|  | HC  (n=74 | PD+DA (n=151) | PD-DA (n=71) | Dystonia (n=82) | F value | P value^1^ | P value^1^  HC vs PD+DA | P value^1^  HC vs  PD-DA | P value^1^  HC vs Dys | P value^1^  PD+DA vs  PD-DA | P value^1^  PD+DA vs Dys | P value^1^  PD-DA vs Dys |
| --- | --- | --- | --- | --- | --- | --- | --- | --- | --- | --- | --- | --- |
| **Age** | 63.69 (12.36) | 65.33 (8.35) | 67.93 (8.92) | 54.80 (12.49) | 25.09 | <**0.001^2^** | 1.00^2^ | 0.88^2^ | **<0.001^2^** | 0.51^2^ | **<0.001^2^** | **<0.001^2^** |
|  |  |  |  |  |  |  |  |  |  |  |  |  |
| **Any ICD** | 13 (17.6) | 40 (26.5) | 10 (14.1) | 17 (20.7) |  | 0.16 |  |  |  |  |  |  |
| **Multiple ICDs** | 4 (5.4) | 9 (6.0) | 3 (4.3) | 3 (3.7) |  | 0.91 |  |  |  |  |  |  |
| Gambling | 1 (1.4) | 6 (4.0) | 3 (4.2) | 1 (1.2) |  | 0.53 |  |  |  |  |  |  |
| Sex | 1 (1.4) | 9 (6.0) | 3 (4.2) | 2 (2.5) |  | 0.38 |  |  |  |  |  |  |
| Shopping | 5 (6.8) | 15 (9.9) | 6 (8.6) | 8 (9.8) |  | 0.90 |  |  |  |  |  |  |
| Eating | 11 (14.9) | 20 (13.3) | 2 (2.9) | 9 (11.0) |  | 0.05 |  |  |  |  |  |  |

**^1^**Fisher’s exact test, **^2^**One-way ANOVA with Bonferroni correction

ICD = Impulse control disorder, HC = Healthy controls, PD+DA = Parkinson’s disease with dopamine agonist medication,

PD-DA = Parkinson’s disease without dopamine agonist medication

Note that the number of subjects included in each analysis varies according to how many subjects provided sufficient data.

**Supplementary table 4. Only men (n=336). The values are mean (SD) or n (%).**

|  | HC  (n=45) | PD+DA (n=166) | PD-DA (n=110) | Dystonia (n=15) | F value | P value^1^ | P value^1^  HC vs PD+DA | P value^1^  HC vs  PD-DA | P value^1^  HC vs Dys | P value^1^  PD+DA vs  PD-DA | P value^1^  PD+DA vs Dys | P value^1^  PD-DA vs Dys |
| --- | --- | --- | --- | --- | --- | --- | --- | --- | --- | --- | --- | --- |
| **Age** | 67.73 (11.81) | 65.34 (8.08) | 70.38 (6.69) | 60.73 (11.80) | 10.85 | **<0.001^2^** | 0.56 ^2^ | 0.47^2^ | **0.035^2^** | **<0.001**^2^ | 0.27^2^ | **<0.001^2^** |
|  |  |  |  |  |  |  |  |  |  |  |  |  |
| **Any ICD** | 13 (28.9) | 67 (40.4) | 37 (33.6) | 3 (20.0) |  | 0.25 |  |  |  |  |  |  |
| **Multiple ICDs** | 2 (4.4) | 22 (13.3) | 13 (12.1) | 2 (13.3) |  | 0.40 |  |  |  |  |  |  |
| Gambling | 1 (2.2) | 11 (6.6) | 5 (4.6) | 1 (6.7) |  | 0.64 |  |  |  |  |  |  |
| Sex | 8 (17.8) | 54 (32.5) | 29 (26.6) | 3 (20.0) |  | 0.22 |  |  |  |  |  |  |
| Shopping | 5 (11.1) | 11 (6.6) | 6 (5.5) | 1 (6.7) |  | 0.57 |  |  |  |  |  |  |
| Eating | 3 (6.7) | 17 (10.2) | 11 (10.1) | 1 (6.7) |  | 0.94 |  |  |  |  |  |  |

**^1^**Fisher’s exact test, **^2^**One-way ANOVA with Bonferroni correction

ICD = Impulse control disorder, HC = Healthy controls, PD+DA = Parkinson’s disease with dopamine agonist medication,

PD-DA = Parkinson’s disease without dopamine agonist medication

Note that the number of subjects included in each analysis varies according to how many subjects provided sufficient data.
